# The 2022 Academy for Multidisciplinary Neurotraumatology (AMN) Virtual Congress – Day 1

**DOI:** 10.25122/jml-2022-1009

**Published:** 2022-07

**Authors:** Stefana-Andrada Dobran, Alexandra-Mihaela Gherman, Dafin Fior Muresanu

**Affiliations:** 1. RoNeuro Institute for Neurological Research and Diagnostic, Cluj-Napoca, Romania; 2. Sociology Department, Babes-Bolyai University, Cluj-Napoca, Romania; 3. Department of Neuroscience, Iuliu Hatieganu University of Medicine and Pharmacy, Cluj-Napoca, Romania

## INTRODUCTION

The 20^th^ Congress of the Academy for Multidisciplinary Neurotraumatology (AMN) from 2022, taking place online between May 12^th^ and 13^th^, revealed an awe-inspiring incursion into the complex subject of neurotrauma. Traumatic Brain Injury (TBI) represents a major medical, healthcare, and socioeconomic problem worldwide and one of the most important causes of disability and mortality. Over 700 participants from various countries attended the much-expected educational event, which aimed to set the stage for lively debates on a plethora of aspects under the broad domain of neurosciences. Thrilling lectures and presentations took place over two days and the event included 6 sessions and 4 interactive panels held by 26 speakers.

The event brought together a lively audience with diverse backgrounds, such as neurology, psychology, neurosurgery, neurorehabilitation, psychiatry, psychotherapy, and public health. The Congress was opened by the steering committee, which included Klaus von Wild (Germany), Dafin F. Muresanu (Romania), Johannes Vester (Germany), Nicole von Steinbüchel (Germany), and Volker Hömberg (Germany).

## CONGRESS SESSIONS

### Session 1

Session one introduced the audience to the topic of TBI research and was structured into two presentations. The former, by Johannes Vester (Germany), the AMN president and Associate Professor at the Department of Neuroscience from Iuliu Hatieganu University of Medicine and Pharmacy in Cluj-Napoca (Romania), was centered around the multidimensional approaches in TBI research and presented insights from the Cerebrolysin Asian Pacific Trial in Acute Brain Injury and Neurorecovery (CAPTAIN) I and II trials. Moreover, Prof. Vester discussed the limitations of the dichotomization of results, of binary thinking in TBI research, and the importance of full-scale analysis.

The second presentation, by Volker Hömberg (Germany), Chairman of AMN Scientific Program Committee and President-Elect of the World Federation for Neurorehabilitation (WFNR), approached the vast and complex subject of neurorehabilitation, discussing future approaches. Neurorehabilitation of TBI represents a vast topic, ranging from early rehabilitation of patients to support and reintegration into the social and professional environment. Therefore, discussing solutions for neurorehabilitation is of utmost importance, especially considering the gap experienced in low- and middle-income countries (LMIC) with regard to meeting the needs of the patients, as well as the disruption caused by the COVID-19 pandemic. Prof. Hömberg discussed the role of high-tech options (*e.g*. Intelligent Mechanical Training Devices, Gaming Technology for motor and cognitive training, Brain-Computer Interfaces, Neuromodulation) in contrast to low-tech options (learning-based coaching strategies and procedures, community-based and caregiver support options, self-training strategies), comparing their limitations and benefits. Furthermore, Prof. Hömberg offered insight into the role of digital communication strategies in allowing the dissemination of knowledge and skills to larger populations.

Prof. Dafin F. Muresanu, Chairman of the Department of Neurosciences at Iuliu Hatieganu University of Medicine and Pharmacy in Cluj-Napoca (Romania), ended the session with a presentation entitled “New developments in TBI clinical research from a C-RETURN study” highlighting the importance of continuous research in the domain of neurotrauma. Prof. Muresanu suggested that the functional effects of lesions on brain networks require a change in our approach to neurorehabilitation, shifting the emphasis of treatment and restorative trials towards modulating the activity levels in preexisting networks and showcased the role of multimodal drugs in both neuroprotection and neurorecovery. Moreover, Prof. Muresanu discussed the importance of the CAPTAIN trials in TBI research as the first series of trials with multidimensional approaches based on full outcome scales, and the vital role of the C-RETURN study.

### Session 2

The second session, having Prof. Johannes Vester (Germany) and Prof. Andrew Maas (Belgium) as chairpersons, further discussed the role of research in TBI. The session began with a presentation by Andrew Maas (Belgium), Emeritus Professor of Neurosurgery at the Antwerp University Hospital and University of Antwerp (Belgium), on the continuity of care in the trauma chain, presenting the experience of Collaborative European NeuroTrauma Effectiveness Research in TBI (CENTER-TBI). The inspiring CENTER-TBI is a longitudinal prospective study describing Intensive Care Unit (ICU) admission policies, management, and outcome for TBI patients. Prof. Maas showcased the importance of care in the initial phase at the emergency scene and the post-acute care phase. The necessity for the CENTER-TBI study was two-fold, first, the need for improved characterization and classification, and secondly, the issue of the heterogeneity of TBI as a disease, which motivated the search for best practices. Moreover, the importance of guidelines was discussed, as well as the shift towards multimodal approaches in treatment, the disparities in care, and the risk of incomplete recovery following a mild TBI. Lastly, the presentation ended with a statement on the importance of the CENTER-TBI study.

Furthermore, the session was followed by an insight into the CAPTAIN trial from Christian Matula (Austria), Professor and Vice-Chairman at the Department of Neurosurgery from Medical University of Vienna (Austria). In the past years, a few studies significantly impacted the management and outcomes of TBI. The CAPTAIN trial reveals impactful insight into the effect of the pharmacological agent Cerebrolysin. After discussing the neuroprotective and neurorecovery mechanisms of the brain following an acute lesion, he highlighted the importance of neurotrophic factors in the process of protection and recovery. Moreover, Prof. Matula discussed the objectives of the CAPTAIN trial while showcasing the problems of reductionist approaches, and the importance of multidimensional analysis in providing a new direction for clinical and statistical thinking. The results of the trials could be a door opener for future guidelines. Lastly, the grim realities of post-TBI depression encountered in nearly half of all TBI patients were discussed, highlighting the need for proper approaches. As proof of the necessity of multimodal approaches, he ended the presentation with a glimpse into the exciting Neurotrauma Treatment Simulation Center from Vienna, a training program focusing on the patient pathway to recovery.

The final presentation offered an immersive incursion into the tools and instruments necessary for developing and providing the best practices in neurotrauma care by Mark Bayley (Canada), Program Medical Director and Psychiatrist-in-Chief at UHN-Toronto Rehabilitation Institute (Canada), underlining the constant need to improve existing therapies and find new treatments. Prof. Bayley aided the participants in discovering evidence-based resources to support TBI care and rehabilitation, including reviews and clinical practice guidelines, navigating web-based resources developed in Canada, providing demonstrative cases, and, lastly, using the TRICORDRR (Toronto Rehabilitation Institute Concussion Outcome Risk Determination & Rehab Recommendations) tool for the identification of patients most likely to require long-term special care for conclussions.

### Session 3

The first day of the online AMN Congress of 2022 was completed by the 3^rd^ session, having Prof. Nicole von Steinbüchel (Germany) and dr. Juan Carlos Arango-Lasprilla (Spain) as chairpersons. The session centered around neuropsychological aspects of TBI as well as TBI rehabilitation.

Firstly, Nicole von Steinbüchel (Germany), Director of the Institute of Medical Psychology and Medical Sociology of the University Medical Center at Georg-August-University of Goettingen (Germany), outlined the domains and new trends in neuropsychological rehabilitation. The presentation started with an incursion into the past, presenting a reality that remains in place to this day, respectively that only a minority of patients suffering a stroke or traumatic brain injury receive adequate rehabilitation aid. This reality supports the need to enhance and personalize the post-TBI treatment and rehabilitation, especially regarding cognitive function, but also in treating psychiatric, psychological, and daily life problems arising after neurotrauma. With physical treatment receiving more attention in TBI, it is essential to consider the need for proper neurorehabilitation. The sequelae of TBI can be complex and could hamper the reintegration of patients into society, as well as their quality of life. With depression, anxiety, and Post-Traumatic Stress Disorder (PTSD) presented in a significant number of patients early on after a TBI and persisting up to one year, the need for systematic longitudinal evaluation and therapy is apparent. Prof. von Steinbüchel described how neurological rehabilitation aims to improve functions through restitution, substitution, activation, or integration, with each of the above-mentioned requiring specific evaluation and therapeutic strategies in the context of a multidimensional perspective. Recent years have brought the development of new therapeutic technologies, the improvement of neuroimaging techniques, as well as the development and implementation of international multicenter studies to address the gaps in information on neuroimaging and analytic algorithms, the influence of biomarkers on outcomes, the multidimensional outcome assessment, thus paving an encouraging road towards comprehensive neurotrauma care and rehabilitation.

Secondly, Marina Zeldovich (Germany), Dr. rer. nat. at the Institute of Medical Psychology and Medical Sociology in Göttingen (Germany), discussed the results of the CENTER-TBI study on patient-reported and neuropsychological outcomes after TBI. Patients after a TBI often report anxiety, depression, PTSD, post-concussion symptoms, and a decreased quality of life. In addition to clinical diagnosis tools, the patient-reported outcomes offer a more comprehensive image of the subject. To assess modifications in cognitive functions, information on performance-based outcomes provides relevant insight into patient status after a TBI. Dr. Zeldovich presented the findings of the CENTER-TBI studies, highlighting the need for multimodal outcome assessment, the inclusion of health-related quality of life evaluations, and evaluation of cognitive performance.

The presentation was followed by a glimpse into the complexity of rehabilitation after mild traumatic brain injury, by Nada Andelic (Norway), Head of Research and Development at the Department of Physical Medicine and Rehabilitation from University Hospital in Olso (Norway), describing evidence-based recommendations for the treatment of physical, cognitive, and emotional symptoms following a mild TBI. Prof. Andelic familiarized the audience with strategies for treating physical, cognitive, and emotional consequences post mild TBI as well as facilitating the return to pre-morbid functioning. Mild TBI is characterized by the level of consciousness at the time of the injury or half-hour post-injury, along with the presence of other neurological symptoms. Rehabilitation aims to optimize function and reduce disability in patients, as stated by the World Health Organization (WHO). Prof. Andelic pinpointed the effectiveness of rehabilitation efforts in the acute phase, highlighted the importance of comprehensive assessment, and supported the use of biopsychosocial conceptualization in the rehabilitation of persistent symptoms encountered after mild TBI. The importance of contextual factors stems from their interaction and the possibility to negatively or positively impact the outcome and brings forward the need for patient-centered rehabilitation models and evidence-based treatments. Further aspects on neuropsychological rehabilitation, focusing on memory and attention after traumatic brain injury and new technologies were also discussed by Juan Carlos Arango-Lasprilla (Spain), Research Professor at BioCruces Vizcaya Health Research Institute in Bilbao (Spain).

The last presentation of the session approached the topic of neurorehabilitation of psychiatric sequelae after TBI and was conducted by Katrin Rauen (Switzerland), Consultant in Neurology, Psychiatry & Psychotherapy at the Department of Geriatric Psychiatry from University Hospital of Psychiatry Zurich (Switzerland). Nowadays, traumatic brain injury scientifically becomes more important, however, the society is still unaware of its significant medical and healthcare implications and neuropsychiatric repercussions for the patients. Therefore, neuropsychiatric complications are one of the most important factors to consider, as they represent a constant challenge for all medical specialties and can significantly impact the lives of patients and their families. Most TBI patients are seen by GPs (General Practitioners) rather than TBI specialists, underlining the neglected relevance of the disease in the field of Neuropsychiary. Some of the most important aspects to consider are depression, anxiety, fatigue, and insomnia, as well as PTSD and the increased risk of suicide. It is of utmost importance to detect patients with neuropsychiatric sequelae early on and consider the importance of quality-of-life assessment after neurotrauma. The Quality of Life after Brain Injury (QOLIBRI) instrument comes as a solution to this problem. Finally, Dr. Rauen pinpointed strategies for cognitive rehabilitation and the importance of psychiatric diagnosis and rehabilitation in TBI guidelines.

## PANELS

### 1^st^ Panel

The AMN Congress introduced the first Panel, “Research in TBI is full of challenges **–** how to minimize the risks of failure?”, where a respected set of panelists, including Michael Chopp (USA), Karin Diserens (Switzerland), Andrew Maas (Belgium), and Johannes Vester (Germany) had a lively discussion on fitting approaches for the challenges of TBI research. TBI research can be difficult as there is tremendous variability when considering the best approaches. A comprehensive approach should consider the best models, the outcomes and mechanisms, and have a thorough understanding of the selected population. A multidimensional approach from research teams members of varying specialties (neurologists, neurosurgeons, public health specialists, psychologists, *etc*.) could offer a more complex vision of the problems and lead to innovative solutions.

### 2^nd^ Panel

The second Panel, “The incidence of post-TBI cognitive problems is known **–** what should be done to offer effective treatment solutions?”, having Nada Andelic (Norway), Juan Carlos Arango-Lasprilla (Spain), Peter Lackner (Austria), Katrin Rauen (Switzerland), Nicole von Steinbüchel (Germany) and Marina Zeldovich (Germany) as panelists, highlighted approaches to treatment for the cognitive problems resulting from neurotrauma. Cognitive problems are often developed following a TBI and can affect attention, learning, memory, concentration, speech, problem-solving, and planning. As attention is the basis for higher cognitive skills (reasoning, memory), impairment in attention can lead to a wide array of manifestations, from understanding information, following directions, reacting, managing tasks, learning and remembering, making informed decisions, or understanding social boundaries.The impact of the cognitive impairments can reflect significantly on the personal and professional lives of those who have experienced TBI due to difficulty in carrying out executive tasks, distractibility, and difficulty in conversations. The issue of post-TBI cognition is complex and multidimensional, having a substantial impact on the patient and their social circle (family, employers, healthcare providers *etc*.). For this, early identification of the individual at risk and the development of effective treatment solutions, as well as having a follow-up of the patient is of utmost importance.The impact of the cognitive impairments can reflect significantly on the personal and professional lives of those who have experienced TBI due to difficulty in carrying out executive tasks, distractibility, and difficulty in conversations.

The issue of post-TBI cognition is complex and multidimensional, having a substantial impact on the patient and their social circle (family, employers, healthcare providers, *etc*.). For this, early identification of the individual at risk and the development of effective treatment solutions, as well as having a follow-up of the patient is of utmost importance.

